# L-Carnitine Alleviates the Myocardial Infarction and Left Ventricular Remodeling through Bax/Bcl-2 Signal Pathway

**DOI:** 10.1155/2022/9615674

**Published:** 2022-05-23

**Authors:** Hao-Ran Li, Xiao-Ming Zheng, Yan Liu, Jing-Hui Tian, Jie-Jian Kou, Jun-Zhuo Shi, Xiao-Bin Pang, Xin-Mei Xie, Yu Yan

**Affiliations:** ^1^School of Pharmacy, Henan University, Kaifeng 475004, China; ^2^Institute of Chinese Medicine, Henan University, Kaifeng 475004, China; ^3^Department of Nuclear Medicine, The First Affiliated Hospital of Zhengzhou University, Zhengzhou 450052, China; ^4^Department of Pharmacy, Xuchang Central Hospital Affiliated to Henan University of Science and Technology, Xuchang 461000, China; ^5^Department of Pharmacy, Huaihe Hospital of Henan University, Kaifeng 475004, China; ^6^Department of Pharmacy & Institute of Clinical Medical Sciences, China-Japan Friendship Hospital, Beijing 100029, China

## Abstract

**Purpose:**

L-carnitine (LC) is considered to have good therapeutic potential for myocardial infarction (MI), but its mechanism has not been clarified. The aim of the study is to elucidate the cardioprotective effects of LC in mice following MI and related mechanisms.

**Methods:**

ICR mice were treated with LC for 2 weeks after induction of MI with ligation of left anterior descending artery. Electrocardiographic (ECG) recording and echocardiography were used to evaluate cardiac function. H&E staining, TTC staining, and Masson staining were performed for morphological analysis and cardiac fibrosis. ELISA and immunofluorescence were utilized to detect biomarkers and inflammatory mediators. The key proteins in the Bax/Bcl-2 signaling pathway were also examined by Western blot.

**Results:**

Both echocardiography and histological measurement showed an improvement in cardiac function and morphology. Biomarkers such as LDH, NT-proBNP, cTnT, and AST, as well as the inflammatory cytokines IL-1*β*, IL-6, and TNF-*α*, were decreased in plasma of mice receiving LC treatment after myocardial injury. In addition, the expression of *α*-SMA as well as the key proteins in the Bax/Bcl-2 signaling pathway in cardiac myocardium were much lower in mice with LC treatment compared to those without after MI.

**Conclusions:**

Our data suggest that LC can effectively ameliorate left ventricular (LV) remodeling after MI, and its beneficial effects on myocardial function and remodeling may be attributable at least in part to anti-inflammatory and inhibition of the Bax/Bcl-2 apoptotic signaling pathway.

## 1. Introduction

Myocardial infarction (MI) is a disease manifested by cessation of coronary artery blood flow and ischemic necrosis of the myocardium. Left ventricular (LV) remodeling is a disorder followed by the rupture of coronary plaque, the formation of thrombus to block blood vessels, and finally acute myocardial ischemic injury [1, 2]. MI is clinically characterized by significant alteration in ventricular architecture concerning with a series of genome, molecular, cellular, and interstitial changes [3, 4]. In post-acute MI animal models, the process of LV remodeling begins rapidly—usually within the first few hours after an infarct—and continues to progress in a long run. Several studies have demonstrated the point that infarct size is an important determinant of adverse remodeling as larger infarct size is parallel with greater LV remodeling [5–7]. Though mortality reduces significantly due to timely intervention and other effective therapies, prognosis remains poor in patients with large infarction and severe LV dysfunction. Adverse LV remodeling is associated with deterioration of cardiac function and unfavorable outcome such as chronic heart failure, which is one of the most common causes of cardiovascular morbidity and mortality worldwide [8]. To get the situation improved, multifactorial interventions are required to arrest the process of myocardial remodeling.

Cardiac remodeling after MI is a major cause of occurrence and development of congestive heart failure; the severity of remodeling is closely related to the prognosis of heart failure [9]. Although ACEI and *β*-receptor blockers can mitigate the process of remodeling but they cannot completely abrogate the occurrence [10]. Extensive studies have focused on the novel therapeutic options to alleviate the myocardial remodeling and to restore the cardiac function. Metabolic dysfunction and inflammation are regarded as critical factors driving many cardiovascular diseases [11–13]. The correction of the abnormal metabolism via modulation of inflammation seems to be a promising approach against the disease progression. For example, increasing free fatty acid and glucose oxidation with L-carnitine (LC) is shown to improve heart health [3, 14, 15]. LC is a naturally occurring amino acid derivative in human body, which plays an important role in myocardial energy metabolism [16, 17]. It is beneficial not only for the improvement in fatty acid metabolism in cardiomyocytes for energy supply but also for the repair of injured cardiomyocytes. A previous study demonstrated that carnitine administration after acute MI exerts a beneficial effect on LV remodeling, with a significant reduction in the increase in LV volumes in the first year after acute MI [18]. However, the effectiveness and underlying mechanism is still unclear. Hence, we carried out this project to further explore the mechanism and to find potential target for the treatment of post-MI. To this end, the MI model was established with the ligation of left anterior descending branch in mice and effect of LC on inflammation, pathological changes, and possible pathways after myocardial infarction were also investigated to discern the potential targets for the treatment of LV remodeling.

## 2. Materials and Methods

### 2.1. Materials

LC (purity > 98.0%) was obtained from Sigma Technology (Danvers, USA). Biomarkers such as LDH, NT-proBNP, cTnT, and AST, as well as the inflammatory cytokines including IL-1*β*, IL-6 and TNF-*α*, were measured by enzyme-linked immunosorbent assay (ELISA) kits provided by Elabscience Biotechnology Co., Ltd. (Wuhan, China). H&E staining and Masson staining were purchased from Beyotime Biotechnology Co., Ltd. (Shanghai, China). TTC staining was purchased from Sigma Technology (Danvers, USA). Antibodies against Bax (1 : 1000, #2772), Bcl-2 (1 : 1000, #3498), *α*-SMA (1 : 1000, #56856), GAPDH, (1 : 1000, #5174), and cTnT (1 : 1000, #5593) were purchased from Cell Signaling Technology (Shanghai, China).

### 2.2. Animals

A total of 36 male 10-week-old ICR mice weighed 24-26 g were obtained from Beijing Vital River Laboratory Animal Technology. The mice were housed in cages at 22 ± 2°C under a 12 h light/dark cycle with a standard diet and water ad libitum. All animal experiments utilized protocols that were performed according to the Guidelines for the ethical review of laboratory animal welfare People's Republic of China National Standard GB/T 35892-2018 [19] and were approved by the Ethics Committee of Henan University (HUSOM-2019-077).

Mice were randomized into three groups including the LC treatment group after MI (LC), MI group (model), and sham group (sham). 12 mice were assigned to each group. All mice underwent the same permanent coronary artery ligation surgery process, except that the suture placed under the left anterior descending coronary artery was not tied in the sham group. After surgical process, mice receiving daily intraperitoneal with LC (0.5 g/kg) for 2 weeks were assigned to the LC group; mice in the model and sham groups were administered with an equal volume of saline. Two weeks later, all surviving mice underwent echocardiography. The blood samples were subsequently collected to isolate the sera, and the hearts were harvested for Western blot and histological analysis.

### 2.3. Surgical Process

The room temperature was maintained at 25°C. Mice were anesthetized with ether and then fixed in the supine position. The skin of left chest was dissected by a lateral 1.5 cm cut along the left side of the sternum after careful shaving and disinfection. Then, we remove subcutaneous tissues and refract the left pectoralis major muscles. Microretractors were then used to separate the 3rd and 4th ribs enough to get adequate exposure of the operating region, while the ribs were kept intact. The heart was gently squeezed out by pressing the thorax lightly. A 5-0 polypropylene suture was used to suture and ligate at a site about 3 mm from its origin. In the sham group, we performed exactly identical process except that suture pass through the artery did not ligate. After the procedure, the heart was immediately pushed back into the thorax as soon as possible and the chest was closed using suture [20].

### 2.4. Electrocardiographic Recording

Mice were involved in the recording of electrocardiographic (ECG) using the Data Sciences International radiotelemetry data acquisition system (Dataquest A.R.T. version 2.2, Data Sciences International, St Paul, MN, USA). To record the ECG, mice were anesthetized with ether and subsequently transmitter was carefully placed subcutaneously. The two leads of the transmitter were placed as negative lead positioned at the right shoulder while the positive one positioned about 2 cm to the left of the xyphoid process. ECG recording was performed before the surgery as baseline condition, immediately after the surgery and 14 days later after the surgery, respectively.

### 2.5. Echocardiography Evaluation

The mice were subjected to noninvasive transthoracic echocardiography 14 days after the surgery. Echocardiography (echocardiography IE33 ultrasound, S12-4 probe, Philips) was used to assess the extent of LV remodeling and dysfunction. LV dimensions were recorded by a double-blind manner using M-mode images including LV internal dimension at end-diastole (LVIDd), LV internal dimension at end-systole (LVIDs), left ventricular ejection fraction (LVEF), left ventricular fraction shortening (LVFS). Likewise, double-blinded reviewers used Doppler ultrasound to measure the aortic valve peak velocity, heart rate (HR), and stroke volume (SV) and calculate the cardiac output by multiplying the SV and HR [21].

### 2.6. Histological Analysis

Mice were sacrificed, and the heart was quickly removed and acquired from chest cavity. The whole-body weight, heart mass, and left ventricle mass were measured separately. Along the short axis of the heart, we gently sliced the heart into 1.0 mm thick sections. The sections were then incubated with 1% TTC in phosphate solution (pH 7.4) for 15 min at room temperature and then digitally photographed. Next, to assess the remodeling histologically, we performed the fixation, dehydration, and embedding using 4% paraformaldehyde, ethanol, and then paraffin, respectively. LV sections (5 mm) were stained with H&E staining and Masson trichrome.

### 2.7. Enzyme-Linked Immunosorbent Assay

Blood samples were collected via cardiac puncture and centrifuged at 4°C, and the serum obtained was stored at -80°C until further use. The hearts were harvested and washed with normal saline. Heart tissues were homogenized on ice followed by centrifugation, and the supernatant was collected and stored at -80°C before further use. Plasma TNF-*α*, IL-1*β*, IL-6, AST, NT-proBNP, LDH, and cTnT were tested by ELISA following the manufacturer's instructions. Absorbance values were determined by a microplate reader, and sample concentration was calculated using the standard curves.

### 2.8. Immunofluorescence Double Staining

Paraffin sections (8 *μ*m) were dewaxed with xylene and then rehydrated in gradient alcohol. Sections were sealed with 3% H_2_O_2_ and 0.3% Triton-X 100 in PBS for 1 h, and antigen repair was performed with citric acid buffered and blocked with 5% BSA. Then, anti-*α*-SMA and anti-cTnT primary antibodies were incubated overnight at 4°C. The sections were washed with PBS and incubated with a second antibody (anti-mouse or anti-Rabbit) for 2 h. Sections were sealed. Images were then taken using a fluorescence microscope (Nikon, Tokyo, Japan).

### 2.9. Western Blot Analysis

Protein concentrations were measured using a protein assay kit according to the manufacturer's instructions. The proteins in samples were separated by SDS-polyacrylamide gel electrophoresis (PAGE), followed by the transfer into the PVDF membrane. The membrane was incubated with primary antibodies overnight at 4°C and washed and incubated with secondary antibodies for 2 h at room temperature. The immunoblotting images were captured using KODAK image Station 4000R (Carestream Health Inc.). Images were taken and analyzed with ImageJ 2.0.

### 2.10. Statistical Analysis

All data were presented as mean ± standard error of the mean (SEM). Comparison among 3 groups was assessed by one-way ANOVA, followed by Dunnett's post hoc test. *p* < 0.05 was considered statistically significant.

## 3. Results

### 3.1. LC Alleviated Cardiac Function of Post-MI Mice

**Figure 1:**
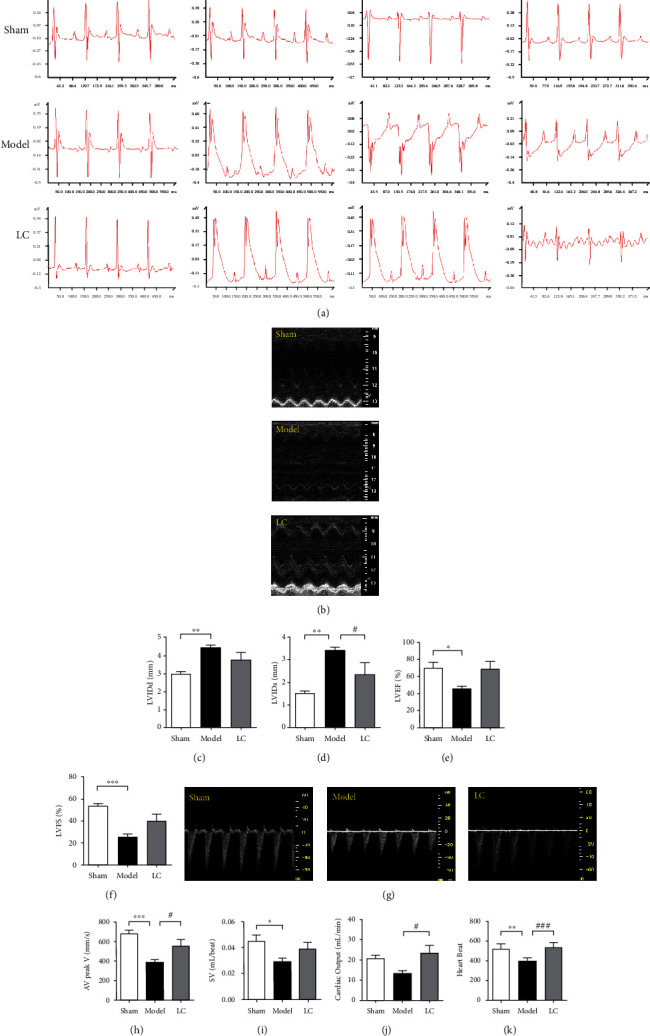
LC alleviated cardiac function of post-MI mice. (a) Representative ECG images of preoperation, postoperation, and 14 days after operation. (b) Representative M-mode tracing obtained at two weeks after surgery. (c) Left ventricular internal diameter at end-diastole (LVIDd). (d) Left ventricular end systolic internal diameter (LVIDs). (e) Left ventricular ejection fraction (LVEF). (f) Left ventricular fraction shortening (LVFS). Data were expressed as means ± SEM. *n* = 5; ^∗^*p* < 0.05, ^∗∗^*p* < 0.01, and ^∗∗∗^*p* < 0.001, compared to the sham group. ^#^*p* < 0.05, compared to the model group. (g) Ventricular tissue Doppler. (h) AV peak V (aortic valve peak velocity). (i) SV (stroke volume). (j) Cardiac output. (k) Heart rate. Values were expressed as means ± SEM. *n* = 5; ^∗^*p* < 0.05, ^∗∗^*p* < 0.01, and ^∗∗∗^*p* < 0.001, compared to the sham group. ^#^*p* < 0.05, ^###^*p* < 0.001, compared to the model group.

The ECG of mice was recorded preoperation, postoperation, and 14 days after operation (ECG was documented via lead I and lead II at day 14 postoperation). It showed that S-T segment was raised after ligation of left anterior descending branch, indicating a successful establishment of MI modeling. After 14 days, compared with the model group, the electrical axis in the LC group was left biased and the S-T segment was partially abolished, suggesting a protective role of LC at chronic stage of MI (Figure 1(a)). To further assess the role of LC in restoring cardiac function, representative echocardiography images from 3 groups were shown in Figure 1(b), and the MI model group had an enlargement of the left ventricles as indicated by an increase in LVIDd, LVIDs compared to those of the sham group. The administration of LC tend to rescue increased LVIDd due to MI (Figure 1(c)) and reversed the increase in LVIDs (*p* < 0.01, Figure 1(d)). In contrast, a significant reduction in LVEF (*p* < 0.05) and LVFS (*p* < 0.001) were also shown in Figures 1(e) and 1(f). There was a tendency towards the improvement of LVEF and LVFS, although both of them did not reach any statistical significance. Doppler ultrasound was also utilized to evaluate the function of left ventricle. In the model group, aortic valve peak velocity and the heart beats were decreased compared to the sham group, which were reversed by LC treatment (Figures 1(h) and 1(k)). A similar pattern was found in stroke volume (Figure 1(i)). Cardiac output also decreased in the model group, and an enhancement was observed after LC treatment (Figure 1(j)). All these findings indicate that LC alleviates the LV remodeling and dysfunction.

### 3.2. LC Reduced the Infarct Size of Post-MI Mice

**Figure 2:**
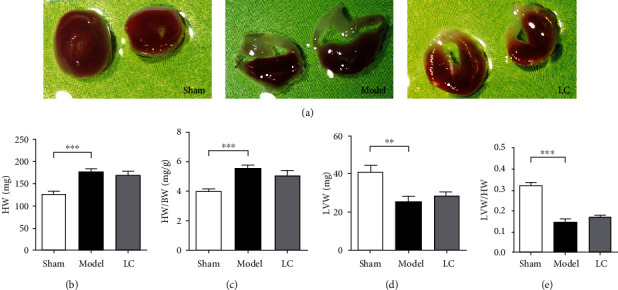
LC reduced the post-MI infarct size. (a) Representative images of TTC staining of cardiac sections from mice. TTC-negative staining area represented for infarct myocardium, while TTC-positive staining area referred to the myocardium that were not affected. (b) Heart weight. (c) The ratio of heart weight to whole body weight. (d) Left ventricular weight. (e) Ratio of left ventricular weight to heart weight. Data were expressed as means ± SEM. *n* = 5; ^∗∗^*p* < 0.01, ^∗∗∗^*p* < 0.001, compared to the sham group.

We further evaluated the effect of LC treatment on cardiac infarct size of mice. The infarct sizes at day 14 post-MI in the model group was obvious when compared to the sham group and LC group exerted a remarkable reduction of infarct size (Figure 2(a)). In addition, the heart weight, the ratio of heart weight to whole-body weight, left ventricular weight, and ratio of left ventricular weight to heart weight all increased significantly in model group, although they were not rescued after LC treatment (Figures 2(b)–2(e)). Our findings suggest that LC could somehow inhibit the left ventricle hypertrophy and help to restore the remolding process.

### 3.3. LC Ameliorated Post-MI Cardiac Hypertrophy and Fibrosis via the Suppression of the Proinflammatory Cytokines

**Figure 3:**
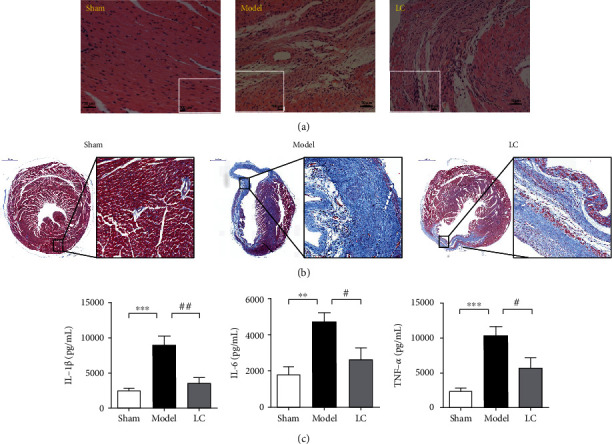
LC suppressed the levels of proinflammatory cytokines and ameliorated cardiac hypertrophy and fibrosis of post-MI mice. (a) Representative image of H&E staining, scale bar = 500 *μ*m in original images and scale bar = 50 *μ*m in enlarged plots. (b) Masson's trichrome-stained heart paraffin sections: collagen was stained in blue and cytoplasm was stained in red, scale bar = 1000 *μ*m in original images and scale bar = 100 *μ*m in enlarged plots. (c) Levels of proinflammatory cytokines were measured after LC treatment. Data were expressed as means ± SEM. *n* = 5; ^∗∗^*p* < 0.01, ^∗∗∗^*p* < 0.001, compared to the sham group. ^#^*p* < 0.05, ^##^*p* < 0.01, compared to the model group.

To evaluate the impact of LC on post-MI cardiac hypertrophy, Masson trichrome staining was performed to assess hypertrophic changes. Masson staining showed that the arrangement of myocardial fibers in the infarcted myocardium was disordered, and there were a lot of blue collagen deposition in the area around the infarct in the model group, which was significantly reduced in the group with LC administration. The left ventricular myocardial longitudinal slices stained with HE showed that the hypertrophy cardiomyopathy was arranged in disorder with interstitial fibrosis. The LC group exhibited an improved condition compared to the model group, suggesting LC is capable of reversing cardiac remodeling (Figures 3(a) and 3(b)). Next, the expression of inflammatory markers such as IL-6, TNF-*α*, and IL-1*β* were under investigation for the potential anti-inflammatory effects of LC therapy. As shown in Figure 3(c), a much higher level of IL-1*β* was displayed in heart tissues of the model group compared to that of the sham group, which was blunted by the LC treatment. A similar pattern was displayed in IL-6 levels. LC treatment could reduce the increase of TNF-*α* caused by MI injuries. The results indicate that LC can inhibit the secretion of inflammation.

### 3.4. LC Inhibited Bax/Bcl-2 Signal Pathway and Suppressed the Biomarkers of Myocardial Injuries

**Figure 4:**
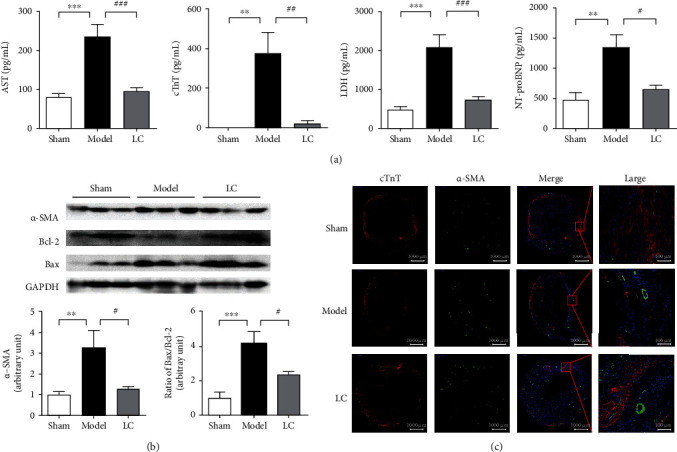
L-carnitine inhibited the Bax/Bcl-2 signal pathway and improved myocardial infarction. (a) Levels of biomarkers for MI were measured after LC treatment. Data were expressed as means ± SEM. *n* = 8; ^∗∗^*p* < 0.01, ^∗∗∗^*p* < 0.001, compared to the sham group. ^#^*p* < 0.05, ^##^*p* < 0.01, and ^###^*p* < 0.001, compared to the model group. (b) Representative Western blots of Bax/Bcl-2 and the level of *α*-SMA in MI mice in the presence or absence of LC treatment. Data were expressed as means ± SEM. *n* = 6; ^∗∗^*p* < 0.01, ^∗∗∗^*p* < 0.001, compared to the sham group. ^#^*p* < 0.05, compared to the model group. (c) Immunofluorescent staining of *α*-SMA (green) and cTnT (red) were performed, scale bar = 1000 *μ*m in original images and scale bar = 100 *μ*m in enlarged plots.

To further investigate the beneficial effect of LC on cardiac function, biomarkers of myocardial injury such as NT-proBNP, cTnT, LDH, and AST in serum were detected. It was found that the activities of NT-proBNP, LDH, cTnT, and AST were increased in the serum in model mice compared with the sham group. Treatment with LC reduced the levels of NT-proBNP, cTnT, LDH, and AST compared with the model group (Figure 4(a)). Western blot results showed an elevated level of *α*-SMA expression in the model group, which was reversed by treatment with LC (*p* < 0.05, Figure 4(b)). An upregulation of Bax expression, downregulation of Bcl-2, and increase of the ratio of Bax to Bcl-2 were displayed in the model group when compared to the sham group (*p* < 0.01, Figure 4(b)). Although LC administration was not able to restore the ratio of Bax to Bcl-2, it did enhance the expression of Bcl-2 compared to that of the model group. Myocardial tissue from the model group had decreased cTnT levels compared to the sham group. There was a tendency towards the elevation of cTnT after LC treatment as revealed by immunofluorescent staining (Figure 4(c)). These results suggest that LC can alleviate myocardial injury.

## 4. Discussion

LC is a naturally occurring bioactive amino acid derivative, which helps to reduce the accumulation of harmful metabolites produced in coronary thrombosis and embolism [22–24]. Studies have found that LC can be used to treat various heart problems, such as myocardial infarction, cardiopulmonary arrest, reperfusion injury, and coronary artery infarction. In fact, previous clinical studies have suggested that LC supplementation can reduce the mortality of myocardial infarction, but the specific mechanism of action is still unclear, and the effect of LC on the apoptosis pathway is still under investigation [4, 25]. In this experiment, the myocardial infarction model was established by ligating the left anterior ligation of mice, and LC was interfered to explore its effect on the level of serum myocardial enzymes in mice with myocardial infarction and further explore the effect and mechanism of LC on myocardial cell apoptosis.

In this study, LC improved post-MI cardiac function as revealed by the ECG finding that the S-T segment of the axis was shifted down in mice receiving LC treatment. LC was also able to improve LV systolic and diastolic function, enhance myocardial output, and reverse LV remodeling via the reduction of myocardial infarct size and suppression of myocardial hypertrophy, The inflammatory levels of TNF-*α*, IL-1*β*, and IL-6 were significantly increased in post-MI mice, which were blunted by LC treatment as well. Moreover, LC treatment can significantly reduce the level of AST, NT-proBNP, cTnT, and LDH in serum and *α*-SMA expression in infarct area.

Most MI are accompanied by ventricular remodeling. Myocardial wall thinning in the infarct area gradually dilates the heart chamber, and in the long-term, there may be ventricular aneurysm formation and myocardial rupture. In addition, due to the large amount of scarring in the infarcted area, the diastolic and systolic dysfunction is caused, which leads to the redistribution of pressure in all directions in the cardiac cavity. Therefore, the remodeling also involved the noninfarcted area, resulting in myocardial hypertrophy in the noninfarcted area, resulting in global enlargement [26–29]. Myocardial fibrosis is an important pathological feature of MI. Myocardial fibrosis can reduce cardiac compliance, affect cardiac systolic and diastolic function, and lead to sudden cardiac death. Reducing myocardial fibrosis can effectively improve cardiac dysfunction and reduce LV remodeling [2, 30]. Of note, myocardial fibrosis in post-MI mice was significantly inhibited and the cardiac function was improved after LC administration.

Inflammation is a very important biological process in the process of many cardiovascular diseases including MI [31–33]. Previous studies have found that a large number of neutrophil infiltration, inflammatory cytokine chemotactic aggregation, and myocardial injury-related proteases appear in the infarcted area and surrounding areas after MI. During the progression of MI, prolonged inflammatory response is further involved in myocardial cell damage in the peri-infarct area, leading to excessive fibrosis and aggravating the progression of ventricular remodeling [17]. Therefore, inhibiting the production of inflammatory factors can slow down the remodeling after MI and reduce the occurrence of heart failure.

Cytosolic enzymes such as AST, NT-proBNP, cTnT, LDH, and *α*-SMA have been used as sensitive markers to assess the severity of MI [34]. cTnT is unique to the myocardium and mainly exists in myofibrillar thin filaments. High levels of cTnT are closely related to myocardial hypoxia, ischemia, necrosis, and cardiac function decline, and the detection of cTnT has the advantages of high sensitivity and specificity, both in the early intervention and prognosis of acute MI have important value [35]. NT-proBNP is a cardiogenic neurohormone synthesized by ventricular myocytes. When the patient's LV pressure load increases and ventricular wall tension increases, the secretion of NT-proBNP will also increase accordingly. The increased level of NT-proBNP indicates that the cardiac insufficiency of patients increased. AST is the main functional enzyme of myocardium, and its activity changes are also closely related to myocardial injury [36, 37]. *α*-SMA is a marker of cardiac fibrosis, and the overexpression of *α*-SMA can promote the growth of atherosclerotic plaques through vascular growth regulation and other pathways [38]. It is further verified that LC can protect the integrity of cardiomyocytes and reduce cardiac burden and myocardial fibrosis, thereby effectively inhibiting the progression of MI.

Apoptosis is a kind of programmed cell suicide, which is dependent on caspase activation and requires energy consumption. It can be regulated by specific genes and can be activated by a series of physiological and pathological factors [39]. The apoptosis of myocardial cells is involved in various cardiovascular diseases including ischemic and nonischemic heart disease, MI, and heart failure. Predisposing factors of myocardial cell apoptosis including angiotensin II, oxygen free radicals, cytokines, and growth factor [40, 41]. Ventricular remodeling is the molecular basis of heart failure. The Bcl-2 family is the first gene family that is found to be involved in the regulation of apoptosis. It includes a number of structurally similar molecules, such as the proapoptotic gene Bax and antiapoptotic gene Bcl-1 and Bcl-2 [42]. Bcl-2 and Bax are regulators of the apoptotic pathway. The changes of Bcl-2 and Bax expressions are an important pathway of myocardial cell apoptosis. And induced apoptosis is considered to be an important pathogenic mechanism of myocardial injury. LC improves MI possibly by inhibiting the Bax/Bcl-2 pathway to reduce apoptosis and cell membrane damage [43].

## 5. Conclusion

LC has a strong protective effect on MI, which is manifested in the reduction of myocardial damage and inflammatory factors, as well as the improvement of LV function. In addition, these effects are partly attributed to the inhibition of Bax/Bcl-2 mediated apoptosis signaling pathway, which has laid a foundation for the study of the mechanism and treatment of MI.

## Data Availability

The datasets used and analyzed during the current study are available from the corresponding author on reasonable request.
